# First occurrence of feline sporotrichosis in a metropolitan area of Central-West Brazil

**DOI:** 10.1590/S1678-9946202466019

**Published:** 2024-04-05

**Authors:** Mariana Almeida Ferreira, Allana de Paula Castilho, Gabrielle Silveira Vargas, Bruna Elisa Patini, André Luís Elias Moreira, Jandra Pacheco dos Santos, Roseli Santos de Freitas Xavier, Gilda Maria Barbaro Del Negro, Carlos Pelleschi Taborda, Isabella Dib Gremião, Álvaro Ferreira

**Affiliations:** 1 Universidade Federal de Goiás, Escola de Veterinária e Zootecnia, Departamento de Medicina Veterinária Preventiva, Goiânia, Goiás, Brazil; 2 Universidade Federal de Goiás, Escola de Veterinária e Zootecnia, Programa de Pós-Graduação em Ciência Animal, Goiânia, Goiás, Brazil; 3 Amparo Vet Hospital Veterinário, Aparecida de Goiânia, Goiás, Brazil; 4 Universidade Federal de Goiás, Instituto de Ciências Biológicas, Laboratório de Biologia Molecular, Goiânia, Goiás, Brazil; 5 Centro Universitário de Goiás, Escola de Medicina Veterinária, Goiânia, Goiás, Brazil; 6 Universidade de São Paulo, Faculdade de Medicina, Instituto de Medicina Tropical de São Paulo, Laboratório de Micologia Médica, São Paulo, São Paulo, Brazil; 7 Universidade de São Paulo, Instituto de Ciências Biomédicas, Departamento de Microbiologia, São Paulo, São Paulo, Brazil; 8 Fundação Oswaldo Cruz, Instituto Nacional de Doenças Infecciosas Evandro Chagas, Laboratório de Pesquisa Clínica em Doenças Zoonóticas dos Animais Domésticos, Rio de Janeiro, Rio de Janeiro, Brazil

**Keywords:** Subcutaneous mycosis, Feline sporotrichosis, *Sporothrix schenckii* complex

## Abstract

Sporotrichosis is a neglected mycosis that affects human and animal hosts, including domestic cats. In Brazil, its most frequently diagnosed etiological agent is *Sporothrix brasiliensis*. Zoonotic transmission of *S. brasiliensis* occurs via direct contact between an infected cat and a susceptible human host. Notification of confirmed cases of feline sporotrichosis is not mandatory in Brazil. The metropolitan area of Goiania city can be considered a silent area for the occurrence of feline sporotrichosis. In this context, voluntary reporting of feline sporotrichosis cases is recommended for all healthcare professionals. This study aimed to report the first occurrence of *S. brasiliensis* in a cat from the metropolitan area of Goiania city. Cytopathology, mycology, thermal dimorphism and calmodulin gene amplification tests were performed. The mycological and molecular biological diagnoses corresponded to *S. brasiliensis*. The etiological agent of zoonotic sporotrichosis was detected in the metropolitan area of Goiania city, and therefore there is a risk of the emergence of new cases of cats infected with *S. brasiliensis* and the occurrence of zoonotic transmission of this fungus.

## INTRODUCTION

Zoonotic sporotrichosis is a serious subcutaneous mycosis caused by *Sporothrix* species that affects mammals^1^. Feline sporotrichosis is caused by *Sporothrix brasiliensis, S*. *schenckii* sensu stricto, *S. globosa* ^2^, *S. humicola* ^3^, and *S. pallida* ^3,4^, but *S. brasiliensis* holds the potential for zoonotic transmission of feline sporotrichosis^5^. *S. brasiliensis* is prevalent in South America, especially in Brazil, where the highest number of cases of feline sporotrichosis has been reported worldwide^6^. *S. brasiliensis* is associated with cat-to-human, cat-to-cat and cat-to-dog transmission^7^, and infection occurs mainly via scratches, bites, or contact with the exudate of cutaneous lesions^8^. The epicenter of this epidemic scenario is the metropolitan area of Rio de Janeiro city, where there has been a high incidence of feline sporotrichosis and zoonotic transmission to humans, which has spread to other states in Brazil^9,10^. Currently, cases of feline sporotrichosis have been reported in most states in the five regions of Brazil^1,10^. On May 25, 2023, the Brazilian Ministry of Health published a technical note^11^ with recommendations for the surveillance of animal sporotrichosis in Brazil, defining silent areas for sporotrichosis as those where transmission to humans and/or animals is unknown. This article reports the first confirmed diagnosis of feline sporotrichosis caused by *S. brasiliensis* in the metropolitan area of Goiania city, Goias State, located in the Central-West region of Brazil.

## CASE REPORT

This report was approved by the Ethics Committee on Animal Use of the Federal University of Goias, under protocol Nº 068/22.

**Figure 1:**
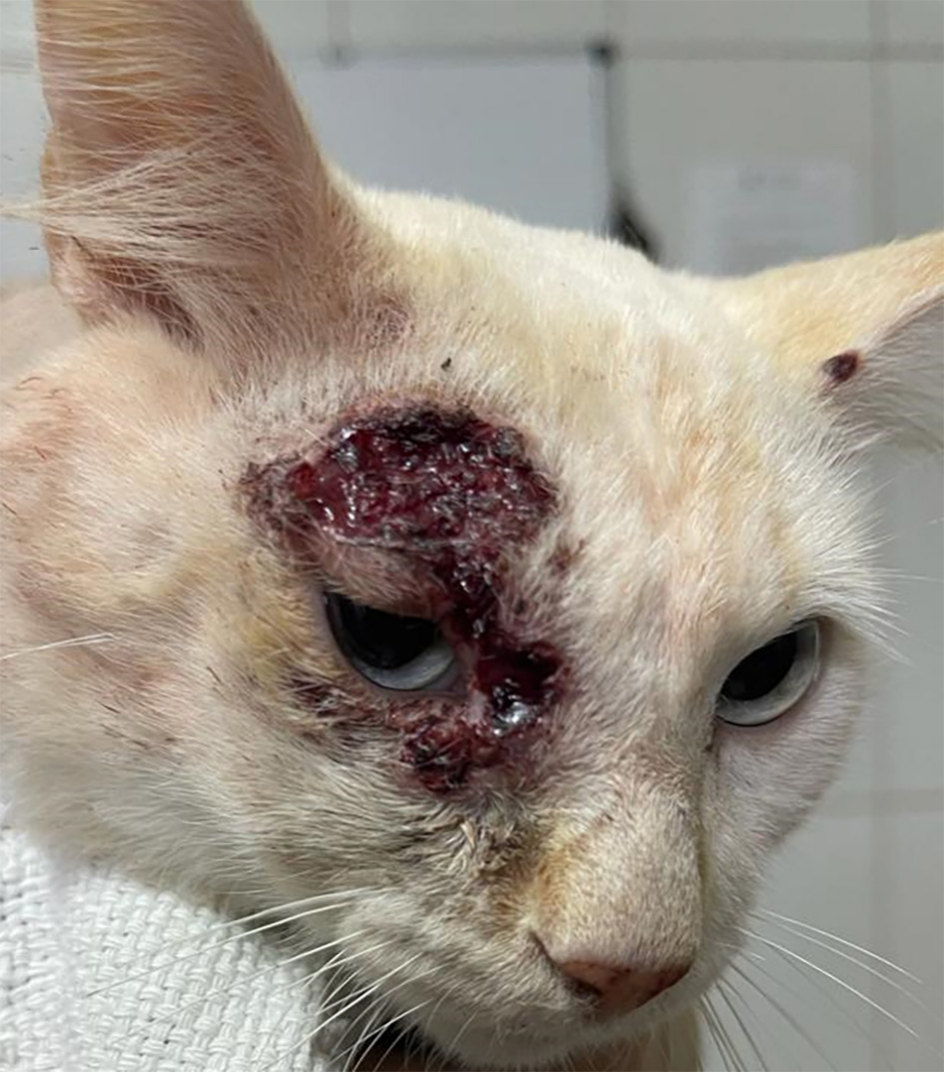
- Multiple ulcerated subcutaneous periocular lesions around the right eye and small ulcerated lesion on the left earlobe.

A one-year-old mixed-breed male cat, with short white coat and weighing 2 kg, was subjected to the trap-neuter-return (TNR) procedure in Aparecida de Goiania city (latitude 16° 49’ 23’’ South and longitude 49° 14’ 32’’ West). A clinical examination revealed multiple periocular ulcerated hemorrhagic skin lesions around the right eye (Figure 1) and a small skin lesion at the left ear margin. Biological samples collected from the lesions were sent to the FungiLab laboratory of the School of Veterinary Medicine of the Federal University of Goias in Goiania city, Goias State, Brazil, and to the Mycology Laboratory of the Institute of Tropical Medicine at the University of Sao Paulo, Sao Paulo State, Brazil. Diagnostic exams included cytology, mycology, thermal dimorphism, and polymerase chain reaction^10,12^. Further tests to evaluate other pathologies could not be performed because the cat died before antifungal therapy could be started. The cause of death of this cat could not be determined. There was no evidence that the cat had bitten or scratched the veterinarian.

**Figure 2:**
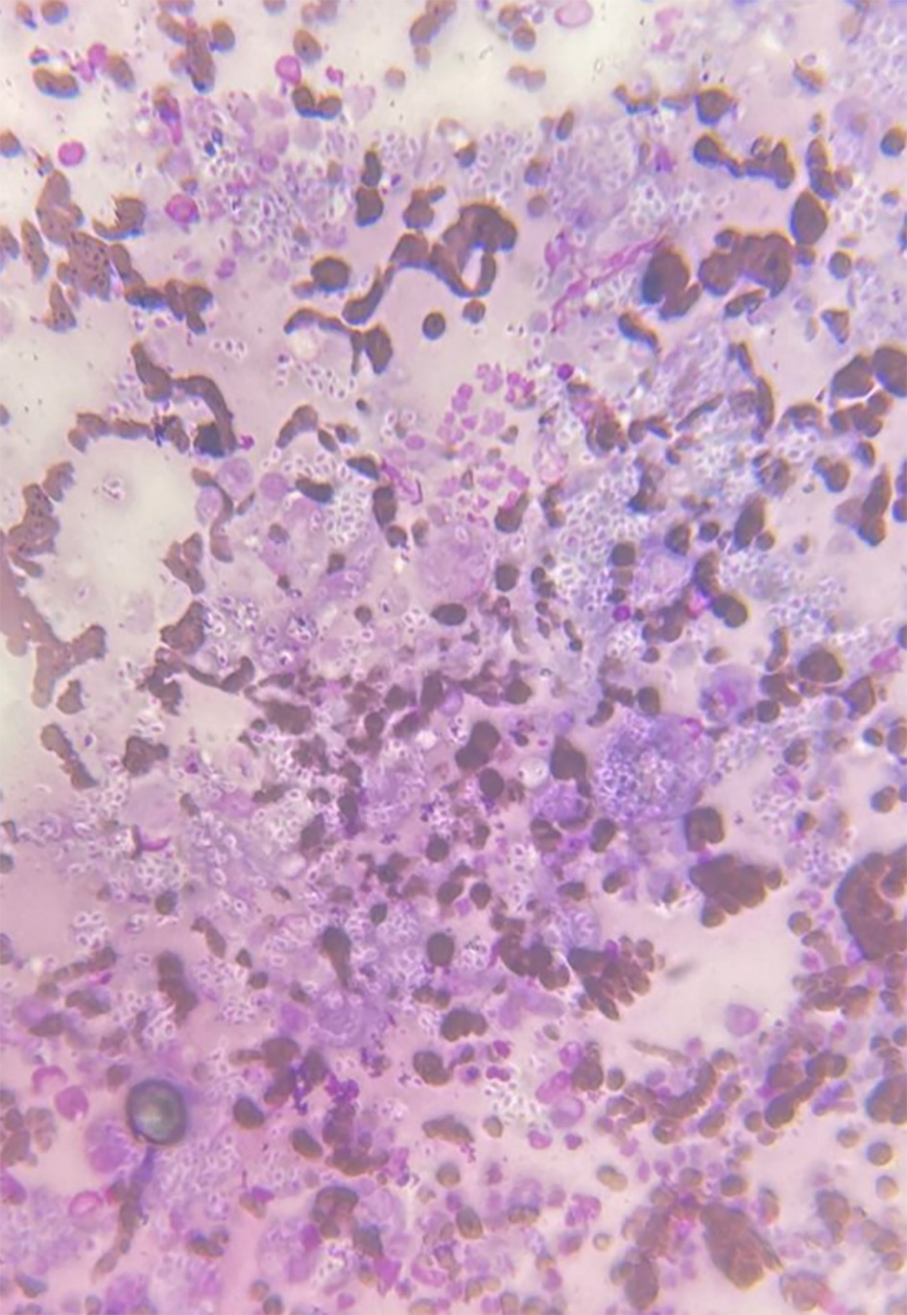
Giemsa-stained extracellular cigar-shaped yeast-like elements at 400x magnification.

**Figure 3:**
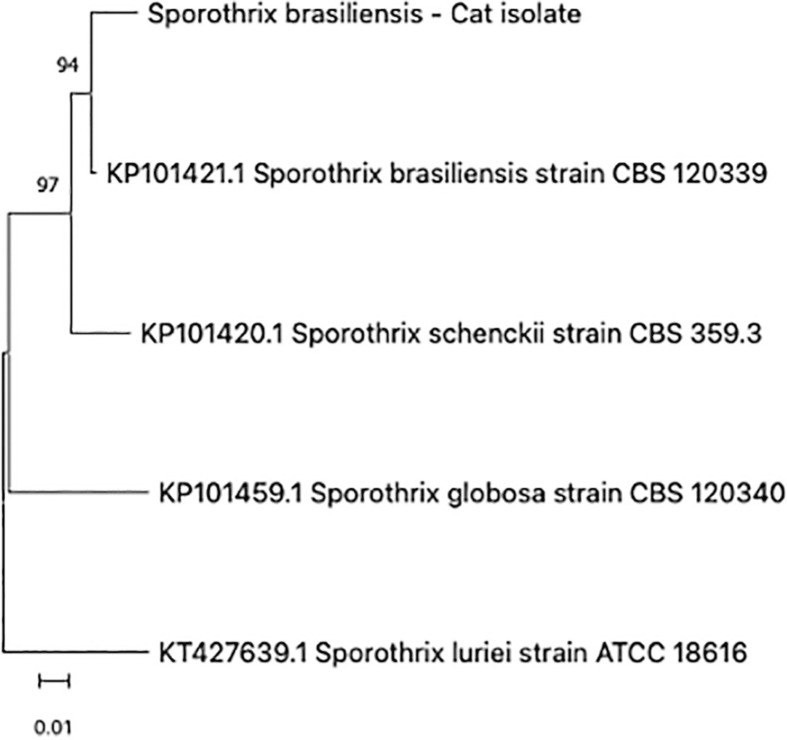
Phylogenetic analysis using the Kimura 2 parameter model with 1000 bootstrap replicates performed in MEGA 11.0, based on the partial calmodulin-encoding gene. The sequence of the feline isolate was compared with the sequences of *S. brasiliensis, S. schenckii* and *S. globosa* reference strains from GenBank.

Cytological examination of the Giemsa-stained material revealed numerous cigar-shaped basophilic yeast elements with a clear border, located within the macrophage cytoplasm and extracellularly, with a presumptive diagnosis of feline sporotrichosis (Figure 2). Exudate from the lesion was then cultured on Mycosel agar at 26 °C for 14 days. The isolated fungus was glabrous, with a velutinous light beige surface and brown color on the underside. The micromorphology of an isolate obtained after 14 days at 26 °C on potato dextrose agar revealed mycelial structures with hyaline, septate and thin hyphae. A conidiogenous cell emerged from the vegetative hyphae at right angles, with teardrop-shaped hyaline conidia emerging from its terminal, forming a rosette-like apical conidial conformation. Pigmented sessile conidia emerged directly from the vegetative hyphae. Thermal dimorphism was confirmed after culturing the mycelium on brain-heart infusion agar (Becton, Dickinson and Company, Heidelberg, Germany) for 14 days at 37 °C in the dark. The identity of the *Sporothrix* isolate was verified by polymerase chain reaction (species-specific PCR)^13^. DNA from the mycelial form of the *Sporothrix* isolate was extracted^13^ and subjected to partial sequencing of the calmodulin-encoding gene^13^, showing 99.5% of similarity to *S. brasiliensis* reference strains deposited in GenBank. A maximum likelihood phylogenetic analysis with 1,000 replication bootstrap was also performed using the MEGA software (version 11.0, Pennsylvania State University, Pennsylvania, USA) (Figure 3)^12,13^. The clinical and laboratory findings confirmed that the pathogenic fungus was *S. brasiliensis*. This case was reported by voluntary notification on the REDCap website of the Brazilian Ministry of Health. This is the first report of *S. brasiliensis* in the metropolitan area of Goiania city, in the Goias State, Brazil.

## DISCUSSION

*Sporothrix brasiliensis* is highly prevalent in Brazil. Epizootics of feline sporotrichosis have been reported in the five regions of Brazil and in most states of the country^10,14^, and this epidemic continues to spread. In South America, cases of feline sporotrichosis have already crossed borders, with *S. brasiliensis* in Argentina and Chile. Moreover, cases of sporotrichosis caused by this fungus have been reported in the United Kingdom after Brazilians traveled to the UK with their cats and cat-to-human transmission occurred^10,15-17^. Garcia Duarte *et al.* ^18^ reported two human cases of *S. brasiliensis* in Paraguay. The authors reported possible dissemination to other countries, as two Brazilians living in Paraguay acquired a cat from a region endemic for *S. brasiliensis* and the cat developed cutaneous sporotrichosis, revealed by classic mycological and histopathological diagnoses. The Goias State is far from the epicenters of sporotrichosis in the country, such as the Rio de Janeiro, Minas Gerais, Sao Paulo, Ceara and Rio Grande do Sul states^10,14^.

Stray cat colonies are common in the metropolitan areas of Brazilian municipalities^9^. The metropolitan area of Aparecida de Goiania has wooded parks and artificial lakes, which are public recreational spaces where people go to walk, play and bring their pets. It should also be noted that human food waste and water sources attract rodents, birds and stray cats^19^, making up the ideal conditions for the cultivation of *Sporothrix*. These include soil pH levels ranging from 3.5 to 9.5, average temperature of 26 °C, relative humidity ranging from 70% to 90%, and plant substrate in decomposition in the soil^19^. The combination of climatic and environmental conditions in the metropolitan area of Goiania city favors the life cycle of *Sporothrix* spp.^19^.

We confirm that cytopathology is an efficient method for screening feline sporotrichosis, as it revealed the presence of numerous cigar-shaped fungal elements and yeasts in the material collected from the cat^8^. The isolation of the pathogen from the collected material was relevant to the diagnosis, as it is the gold standard in sporotrichosis to identify macro- and micromorphological elements compatible with the organism being studied^8^. The calmodulin gene amplification performed in this study confirmed the mycological identification of *S. brasiliensis*.

After the diagnosis of sporotrichosis was made, the owner was contacted and informed that the cat had died, thus precluding the planned antifungal therapy. Animal sporotrichosis is a disease that can be treated with a daily dose of itraconazole (ITZ) alone or in combination with potassium iodide (KI), providing satisfactory therapeutic outcomes^5,8^. However, in cases of zoonotic infection refractory to ITZ plus KI therapy, intralesional liposomal amphotericin B can be used^5,10^. The criteria for cure remain clinical, with remission of all signs and maintenance of therapy for one month after clinical cure^5^. Euthanasia is not the first choice and should only be used when the animal has failed to respond to all therapeutic options and shows irreversible physical deterioration with no chance of recovery. Incineration of fungal elements in animal tissue is recommended to prevent contamination of vegetation and soil^20^.

Subjecting free-roaming cats to the trap-neuter-return procedure provides the opportunity to diagnose animal sporotrichosis in silent areas, as was the case in Aparecida de Goiania city^5^. Continuous education of veterinarians and pet owners can prevent suspicious lesions suggestive of *Sporothrix* spp. and consequently anticipate mycological diagnosis and treatment^5^.

Animal sporotrichosis control programs require integrated actions by public authorities, private veterinary services, public health agencies, and citizens^5^. Mandatory reporting should be established to obtain data on animal sporotrichosis in each region. Given the infrastructural and economic vulnerability of the areas where animal sporotrichosis is commonly detected, free diagnosis and treatment would be the ideal way to contain the spread of this epidemic^20^. *Sporothrix brasiliensis* is an emerging public health threat in Brazil^7^, and these findings should serve to sensitize veterinarians, cat owners, and public health agencies to prevent an outbreak of feline sporotrichosis in the Goias State, Brazil.

## CONCLUSIONS

The fungus *S. brasiliensis* is currently circulating in the metropolitan area of Goiania city, Goias State, Brazil, and causing sporotrichosis in infected free-roaming cats in this region. A disease control program involving integrated actions by public authorities, private veterinary services, public health agencies, and citizens is needed to prevent the spread of this zoonosis.
